# The relationship between quality of life (EORTC QLQ-C30) and survival in patients with gastro-oesophageal cancer

**DOI:** 10.1038/sj.bjc.6604248

**Published:** 2008-02-12

**Authors:** M McKernan, D C McMillan, J R Anderson, W J Angerson, R C Stuart

**Affiliations:** 1 University Department of Surgery, Royal Infirmary, Glasgow G31 2ER, UK; 2 Department of Surgery, Southern General Hospital, Glasgow G51, UK

**Keywords:** gastro-oesophageal cancer, stage, treatment, quality of life

## Abstract

It remains unclear whether any aspect of quality of life has a role in predicting survival in an unselected cohort of patients with gastro-oesophageal cancer. Therefore the aim of the present study was to examine the relationship between quality of life (EORTC QLQ-C30), clinico-pathological characteristics and survival in patients with gastro-oesophageal cancer. Patients presenting with gastric or oesophageal cancer, staged using the UICC tumour node metastasis (TNM) classification and who received either potentially curative surgery or palliative treatment between November 1997 and December 2002 (*n*=152) participated in a quality of life study, using the EORTC QLQ-C30 core questionnaire. On univariate analysis, age (*P*<0.01), tumour length (*P*<0.0001), TNM stage (*P*<0.0001), weight loss (*P*<0.0001), dysphagia score (*P*<0.001), performance status (*P*<0.1) and treatment (*P*<0.0001) were significantly associated with cancer-specific survival. EORTC QLQ-C30, physical functioning (*P*<0.0001), role functioning (*P*<0.001), cognitive functioning (*P*<0.01), social functioning (*P*<0.0001), global quality of life (*P*<0.0001), fatigue (*P*<0.0001), nausea/vomiting (*P*<0.01), pain (*P*<0.001), dyspnoea (*P*<0.0001), appetite loss (*P*<0.0001) and constipation (*P*<0.05) were also significantly associated with cancer-specific survival. On multivariate survival analysis, tumour stage (*P*<0.0001), treatment (*P*<0.001) and appetite loss (*P*<0.0001) were significant independent predictors of cancer-specific survival. The present study highlights the importance of quality of life (EORTC QLQ-C30) measures, in particular appetite loss, as a prognostic factor in these patients.

Gastro-oesophageal cancer is the third commonest cause of cancer death in the UK. Each year, there are approximately 16 500 new cases and over 13 000 deaths attributable to the disease. Overall survival is poor with the majority of patients presenting with advanced, inoperable disease and less than 15% surviving 5 years (Cancer Research UK, Information Resource Centre, 2004). Although there have been improvements in survival following surgery (Ando *et al*, 2000; Hundahl *et al*, 2000; Hofstetter *et al*, 2002; von Rahden and Stein, 2004), for the majority of patients current treatment offers little in terms of improved survival. As a result quality of life in these patients is likely to be of considerable importance (Aaronson, Bullinger and Ahmedzai, 1988; Aaronson *et al*, 1993).

The European Organisation for Research and Treatment of Cancer have developed and validated the EORTC-QLQ-C30 questionnaire designed to assess the quality of life of cancer patients (Aaronson *et al*, 1993). Disease-specific aspects of the questionnaire provide detailed information about the patients' perception of their health. Moreover, it has been reported that, in a few studies, the EORTC QLQ-C30 measurement of quality of life may have prognostic value in patients with gastro-oesophageal cancer (Conroy *et al*, 2006).

Blazeby *et al*, (2001) reported that, in addition to age and TNM stage, physical function or emotional function had independent prognostic value in 92 patients with oesophageal cancer. However, treatment (whether or not the patient underwent surgery) was not included in the model (Blazeby *et al*, 2001).

Fang *et al* (2004) studied 110 patients with squamous oesophageal cancer and concluded that there was evidence to support the correlation of patient-reported quality of life scores with survival; therefore, pretreatment physical functioning might be a surrogate marker of an unrecognised biological prognostic factor. Although performance status was significant on univariate analysis, it was not significant on multivariate analysis; whereas physical functioning was significant (Fang *et al*, 2004).

In contrast, in a study of more than 1000 patients with inoperable gastro-oesophageal cancer, entering three randomised clinical trials, Chau *et al* (2004) reported that no aspect of the QLQ-C30 had independent prognostic value when performance status was considered. However, physical function, role function and global quality of life were associated with survival on univariate analysis. There were no survival differences among patients with oesophageal or gastric cancer (Chau *et al*, 2004). However, this study was retrospective and included selected cohorts of patients.

Therefore, from the above it remains unclear whether any aspect of quality of life other than physical function has a role in predicting survival in an unselected cohort of patients with gastro-oesophageal cancer. The aim of the present study was to examine the relationship between quality of life (EORTC QLQ-C30), clinico-pathological characteristics and survival in patients with gastro-oesophageal cancer.

## PATIENTS AND METHODS

### Patients

Patients presenting with adenocarcinoma or squamous carcinoma of the gastric or oesophageal tract at the Royal Infirmary and Southern General Hospital, Glasgow between November 1997 and December 2002 (*n*=152) participated in a quality of life study, using the EORTC QLQ-C30 core questionnaire.

The extent of tumour spread was recorded using the TNM 5th edition classification (Sobin and Wittekind, 1997). Tumours around the gastro-oesophageal junction were further classified according to tumour site, using the Siewert system; type 1 and 2 lesions of the gastro-oesophageal junction were designated as cancers of the oesophagus. Type 3 tumours of the cardia were designated as gastric cancers (Siewert and Stein, 1998).

For gastric cancers, TNM stage I–III tumours were considered to be potentially amenable to curative surgical resection. For oesophageal cancers, TNM stage I–III tumours, excluding T4, were deemed to be potentially amenable to curative surgical resection. Patients who had stage 1 and 2 disease but whose performance status was poor or who had significant comorbidity were deemed not suitable for surgery and went forward for active palliative treatment or supportive care. There were 152 patients included in the study, 69 patients underwent surgery and 83 patients received active palliative treatment or supportive care.

The study was approved by the Research Ethics Committee of the Royal Infirmary and Southern General Hospital, Glasgow.

### Methods

Clinical and demographic variables were recorded at the patient's initial presentation and included age, sex, tumour type, site and length, TNM stage, ECOG performance status, weight loss and dysphagia.

Following diagnosis but prior to treatment the lead clinician approached patients as to whether they would participate in a study to examine their quality of life. If they gave informed consent they were given the EORTC QLQ-C30 questionnaire to complete.

Different aspects of quality of life were assessed using this cancer-specific 30-item questionnaire, which has six functional scales (physical, role, emotional, cognitive, social, global health status) and several questions relating to a range of physical symptoms (Aaronson *et al*, 1993). Patients marked to what extent each statement applied to them. A number of patients were excluded because they were unlikely to understand the questionnaire either due to language, brain metastases, delirium or confusion. Neither age nor performance status were considered when offering the patient questionnaire. Few subjects were excluded (less than 10 patients) and therefore in those patients offered the questionnaire the bias was likely to be small.

### Statistics

Scoring algorithms have been produced by the EORTC Quality of Life Study Group. The sum of items in each category is added and the total divided by the number of questions in the category. A linear transformation is then undertaken to convert this to a percentage scale with a higher score representing a higher response level. Thus a high score for functional scale represents a high/healthy level of functioning. A high score for the global health status/quality of life represents a high quality of life. In contrast, a high score for the symptom scale represents a higher level of symptoms/problems (Aaronson *et al*, 1993).

Data are presented as the median and range. Survival was determined from the time of biopsy proven diagnosis, and the end point for survival analysis was cancer-specific death. Patients were followed up at their clinic or endoscopy appointments and information on date and cause of death was checked with that received by the cancer registration system through the Registrar General (Scotland). Deaths up to the end of April 2007 were included in the analysis.

Univariate and multivariate survival analysis and calculation of hazard ratios (HR) were performed using a Cox regression model. For simplicity of presentation, a single hazard ratio was calculated for each ordered categorical variable, corresponding to the relative risk between adjacent categories. Hazard ratios for EORTC quality of life and symptom scores relate to a one percentage point increase in the score. Owing to the large number of covariates examined, only those that were significant on univariate analysis were included in the multivariate analysis, and only main effects were considered. The analysis was performed using a backward stepwise procedure to derive a final model of the variables that had a significant relationship with survival. To remove a variable from the model, the corresponding *P*-value had to be greater than 0.05. The proportional hazards assumption was checked using log minus log plots.

Comparison of the association between tumour site, TNM stage, treatment and the functional (physical, role, emotional, cognitive, social, global health status) and physical symptoms (fatigue, pain and appetite loss) scales of the EORTC-QLQ-C30 quality of life questionnaire was carried out using the *χ*^2^-test or Mann–Whitney *U*-test where appropriate. Analysis was performed using SPSS software (SPSS Inc. Chicago, IL, USA).

## RESULTS

Patient characteristics and cancer specific survival analysis of patients with gastro-oesophageal cancer (*n*=152) are shown in Table 1. The minimum follow-up period was 54 months or until date of death, the median follow-up for survivors was 81 months, one patient was lost to follow up and one patient withdrew from the study. During this period 106 (70%) patients died from their disease and 14 (9%) died from comorbid disease.

The majority of patients were over the age of 65 years (57%), male (68%) and had adenocarcinomas (84%). The majority of patients presented with weight loss (66%), had little or no dysphagia and a near normal performance status (ECOG-ps, 71%). The majority of patients had EORTC QLQ-C30 function scores above 50 (physical functioning 100%, role functioning 65%, emotional functioning 74%, cognitive functioning 83%, social functioning 79% and global quality of life 56%) and symptom scores below 50 (fatigue 69%, nausea/vomiting 85%, pain 86%, dyspnoea 79%, sleep disturbance 69%, appetite loss 64%, constipation 76%, diarrhoea 95% and financial difficulties 89%) and therefore had apparently normal quality of life (Table 1).

On univariate analysis, age (*P*<0.01), tumour length (*P*<0.0001), TNM stage (*P*<0.0001), weight loss (*P*<0.0001), dysphagia score (*P*<0.001), performance status (*P*<0.1) and treatment (*P*<0.0001) were significantly associated with cancer-specific survival. EORTC QLQ-C30, physical functioning (*P*<0.0001), role functioning (*P*<0.001), cognitive functioning (*P*<0.1), social functioning (*P*<0.0001), global quality of life (*P*<0.0001), fatigue (*P*<0.0001), nausea/vomiting (*P*<0.01), pain (*P*<0.001), dyspnoea (*P*<0.0001), appetite loss (*P*<0.0001) and constipation (*P*<0.01) were also significantly associated with cancer-specific survival.

**Figure 1:**
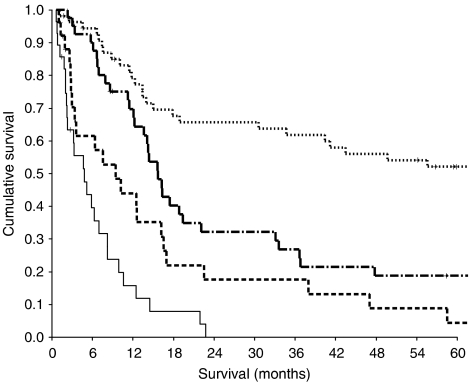
The relationship between appetite loss (None, A little, Quite a bit, Very much, from top to bottom) and cancer specific survival in patients with gastro-oesophageal cancer.

On multivariate analysis, tumour stage (*P*<0.001), treatment (*P*<0.0001) and appetite loss (*P*<0.0001) were significantly independent predictors of cancer-specific survival. The relationship between appetite loss and cancer-specific survival in patients with gastro-oesophageal cancer is shown in Figure 1.

When appetite loss was rescaled so that the four categories were represented by an integer score of 0 to 3 (rather than a percentage score), the unadjusted hazard ratio comparing adjacent categories was 2.06 (95% CI 1.72–2.48, *P*<0.0001). When adjusted for stage and treatment, it was 1.72 (95% CI 1.41–2.08, *P*<0.0001). When adjusted for stage, treatment and remaining clinico-pathological variables, it was 2.07 (95% CI 1.61–2.67, *P*<0.0001). When adjusted for stage, treatment, remaining clinico-pathological variables and quality of life and symptom scores, it was 2.03 (95% CI 1.40–2.94, *P*=0.0002).

In the present study C-reactive protein concentrations, at the time of quality of life assessment, were available in 94 patients (57 patients <10 mg l^−1^, 37 patients >10 mg l^−1^) and were significantly associated with poorer cancer-specific survival (*P*<0.0001). Therefore we included C-reactive protein in addition to TNM stage, treatment and appetite loss in the multivariate survival model. TNM stage (HR 1.37, 95% CI 1.01–1.87, *P*=0.0426), treatment (HR 3.67, 95% CI 1.74–7.75, *P*=0.0006), appetite loss (HR 1.02, 95% CI 1.01–1.03, *P*<0.0001) and C-reactive protein (HR 2.15, 95% CI 1.21–3.83, *P*=0.0091) were independently associated with cancer-specific survival.

The relationship between tumour site, clinico-pathological characteristics and quality of life in patients with gastro-oesophageal cancer is shown in Table 2. Compared with the gastric cancer patients, oesophageal cancer patients were older (*P*<0.01), had more dysphagia (*P*<0.001) and a poorer ECOG-ps (*P*<0.05). In terms of quality of life, compared with the gastric cancer patients, oesophageal cancer patients had higher emotional functioning (*P*<0.01), cognitive functioning (*P*<0.05), less nausea and vomiting (*P*<0.05).

The relationship between TNM stage and clinico-pathological and quality of life characteristics in patients with gastric-oesophageal cancer is shown in Table 3. With increasing TNM stage patients had greater weight loss (*P*<0.01) and were less likely to have had surgery (*P*<0.001). In terms of quality of life, with increasing TNM stage there was poorer physical functioning (*P*<0.05), emotional functioning (*P*<0.05), social functioning (*P*<0.01) and global quality of life (*P*<0.01). In terms of symptoms, with increasing TNM stage there was more fatigue (*P*<0.01), appetite loss (*P*<0.001), dyspnoea (*P*<0.05) and constipation (*P*<0.05).

The relationship between appetite loss, clinico-pathological characteristics and quality of life in patients with gastric-oesophageal cancer is shown in Table 4. Increasing appetite loss was associated with greater tumour length (*P*<0.05), TNM stage (*P*<0.001) and the operability of the tumour (*P*<0.001). Also, increasing appetite loss was associated with weight loss (*P*<0.001) and dysphagia (*P*<0.001). In terms of quality of life, increasing appetite loss was associated with poorer physical (*P*<0.001), role (*P*<0.001), emotional (*P*<0.01), cognitive (*P*<0.01), social (*P*<0.001) and global quality of life (*P*<0.001) functioning. In terms of symptoms, with increasing appetite loss there was more fatigue (*P*<0.01), nausea and vomiting (*P*<0.001), pain (*P*<0.001), sleep disturbance (<0.05) and constipation (*P*<0.001).

The relationship between systemic inflammatory response, as evidenced by elevated C-reactive protein, clinico-pathological and quality of life characteristics in patients with gastric-oesophageal cancer is shown in Table 5. An elevated C-reactive protein was associated with greater tumour length (*P*<0.01), advanced TNM stage (*P*<0.01) and the operability of the tumour (*P*<0.001) and a poorer ECOG-ps (*P*<0.05). In terms of quality of life, an elevated C-reactive protein was associated with poorer physical (*P*<0.01), role (*P*<0.05) and social (*P*<0.05) functioning. In terms of symptoms, with an elevated C-reactive protein was associated with more fatigue (*P*<0.01), pain (*P*<0.05) and appetite loss (*P*<0.01).

## DISCUSSION

In the present study tumour site was not associated with major differences in EORTC QLQ-C30 quality of life function or symptom scores. However, there were major differences in quality of life and symptom scores with increasing stage of disease. In particular, social functioning, fatigue, appetite loss and global quality of life were all impaired with increasing tumour stage.

As might be expected in view of these associations with tumour stage, the majority of quality of life and symptom scores predicted survival on univariate analysis. It was of interest, however, that appetite loss remained an independently significant prognostic factor even after adjustment for TNM stage and treatment. Furthermore, the predictive value of appetite loss was maintained even after adjustment for all other clinico-pathological variables and quality of life and symptom scores. Taken together the results of the present study highlight the importance of appetite loss as a presenting symptom in patients with gastro-oesophageal cancer.

Few studies have examined the relationship between aspects of quality of life and survival in patients with gastro-oesophageal cancer. The results of the present study are consistent with the report of Fang *et al* (2004) who reported that appetite loss was associated with poorer survival in 110 patients with oesophageal cancer. However, the association was much weaker than that of the present study and was not significant in multivariate analysis. Furthermore, the follow-up period and the number of patients who died of their disease were not defined. Blazeby *et al* (1995), in a smaller study of 59 patients with oesophageal cancer, also reported that appetite loss was associated with poorer survival.

The basis of the relationship between appetite loss and poorer cancer-specific survival cannot be determined by the present cross sectional study. However, it was of interest that appetite loss was closely associated with nausea and vomiting, dysphagia and weight loss and therefore it may be that these symptoms result in appetite loss and the consequent loss of weight, which has long been recognised to impact on outcome (Dewys *et al*, 1980).

A number of workers have implicated the systemic inflammatory response in this process (Kotler, 2000; MacDonald, 2007). O'Gorman *et al* (1988), in a cross sectional study, showed that in addition to appetite loss and weight loss, the systemic inflammatory response was an important factor in determining patients' quality of life (EORTC QLQ-C30) in gastro-intestinal cancer patients (O'Gorman *et al*, 1988). Therefore, it is of interest that two recent studies have shown that the presence of a systemic inflammatory response, as evidenced by an elevated C-reactive protein, predicts survival in both operable (Crumley *et al*, 2006a) and inoperable (Crumley *et al*, 2006b) gastro-oesophageal cancer patients. In the present study C-reactive protein concentrations, at the time of quality of life assessment, were available in 94 (62%) patients. Consistent with previous work an elevated C-reactive protein concentration was associated with increased appetite loss and when included in the multivariate analysis, an elevated C-reactive protein concentration was independently associated with poorer cancer-specific survival. However, even those patients without an elevated C-reactive protein concentration reported some appetite loss and the independent prognostic value of appetite loss remained, thus confirming the importance of appetite loss in the multifactorial nature of weight loss and poor outcome in these patients (MacDonald, 2007).

In summary, in patients with gastro-oesophageal cancer, routinely used prognostic factors are based predominantly on clinical and pathological findings. The present study highlights the importance of quality of life (EORTC QLQ-C30) measures, in particular appetite loss, as prognostic factors in these patients.

## Figures and Tables

**Table 1:** The relationship between clinico-pathological characteristics, quality of life and cancer-specific survival in patients with gastro-oesophageal cancer (*n*=152)

	**Patients (*n*=152)**	**Univariate analysis HR (95%CI)**	***P*-value**	**Multivariate analysis HR (95%CI)**	***P*-value**
Age: (<65/65–74/⩾75)	66/56/30	1.46 (1.14–1.89)	0.0033		
Sex: (male/female)	104/48	0.84 (0.55–1.30)	0.4377		
Tumour type: (adeno/squamous)	127/25	1.40 (0.83–2.36)	0.2016		
Tumour site: (oesophagus/gastric)	70/82	0.88 (0.60–1.29)	0.5163		
Tumour length: (<5/510/>10 cm)	60/70/12	2.37 (1.71–3.27)	<0.0001		
TNM stage: (I/II/III/IV)	28/46/34/41	2.29 (1.84–2.83)	<0.0001	1.65 (1.25–2.18)	<0.0004
Weight loss: (no/yes)	51/101	3.08 (1.94–4.89)	<0.0001		
Dysphagia score: (1/2/3/4/5)	81/23/32/15/1	1.37 (1.16–1.63)	0.0003		
ECOG: (0–1/2/3–4)	108/38/6	1.61 (1.14–2.27)	0.0069		
Treatment: (operable/inoperable)	69/83	8.12 (5.06–13.03)	<0.0001	5.29 (2.80–9.97)	<0.0001
					
EORTC QLQ-C30 (0–100)	Median (range)				
Physical functioning	93 (66.7–100)	0.96 (0.94–0.98)	0.0001		
Role functioning	66.7 (0–100)	0.99 (0.99–1.00)	0.0006		
Emotional functioning	66.7 (0–100	1.00 (0.99–1.00)	0.1302		
Cognitive functioning	83.3 (0–100)	0.99 (0.98–0.99)	0.0051		
Social functioning	83.3 (0–100)	0.99 (0.98–0.99)	<0.0001		
Global quality of life	50 (0–100)	0.98 (0.97–0.99)	<0.0001		
Fatigue	33.3 (0–100)	1.02 (1.01–1.02)	<0.0001		
Nausea and vomiting	16.7 (0–100)	1.01 (1.00–1.02)	0.0067		
Pain	16.7 (0–100)	1.01 (1.01–1.02)	0.0002		
Dyspnoea	0 (0–100)	1.01 (1.01–1.02)	0.0001		
Sleep disturbance	33.3 (0–100)	1.00 (0.99–1.01)	0.1558		
Appetite loss	33.3 (0–100)	1.02 (1.02–1.03)	<0.0001	1.02 (1.01–1.03)	<0.0001
Constipation	33.3 (0–100)	1.01 (1.00–1.02)	0.0007		
Diarrhoea	0 (0–100)	1.00 (0.99–1.01)	0.9586		
Financial difficulty	0 (0–100)	1.01 (1.00–1.01)	0.0932		

**Table 2:** The relationship between tumour site, clinico-pathological characteristics and quality of life in patients with gastro-oesophageal cancer (*n*=152)

	**Gastric (*n*=82)**	**Oesophageal (*n*=70)**	***P*-value**
Age: (<65 years/ 65–74 years/⩾75 years)	41/29/12	25/27/18	0.0041
Sex: (male/female)	53/29	51/19	0.279
Type: (squamous/adeno)	1/81	24/46	<0.001
Tumour length: (<5 cm/5–10 cm/>10 cm)	33/33/7	27/37/5	0.724
Tumour stage: (I/II/III/IV)	22/13/18/28	6/33/16/13	0.528
Dysphagia score: (1/2/3/4/5)	64/9/8/1/0	17/14/24/1	<0.001
Weight loss: (yes/no)	53/29	48/22	0.610
ECOG: (0–1/2/3–4)	64/17/1	44/21/5	0.018
Treatment: (operable/inoperable)	38/44	31/39	0.800
			
EORTC QLQ-C30 (0–100)	Median (range)	Median (range)	
Physical functioning	93.3 (66.7–100)	93.3 (66.7–100)	0.733
Role functioning	66.7 (0–100)	66.7 (0–100)	0.923
Emotional functioning	66.7 (0–100)	83.3 (0–100)	0.007
Cognitive functioning	83.3 (0–100)	83.3 (0–100)	0.038
Social functioning	83.3 (0–100)	75 (0–100)	0.964
Global quality of life	50 (0–100)	50 (0–100)	0.284
Fatigue	33.3 (0–100)	22.2 (0–100)	0.077
Nausea and vomiting	16.7 (0–100)	0 (0–100)	0.036
Pain	16.7 (0–100)	16.7 (0–100)	0.716
Dyspnoea	33.3 (0–100)	0 (0–100)	0.123
Sleep disturbance	33.3 (0–100)	33.3 (0–100)	0.360
Appetite loss	33.3 (0–100)	33.3 (0–100)	0.624
Constipation	33.3 (0–100)	33.3 (0–100)	0.031
Diarrhoea	0 (0–100)	0 (0–100)	0.802
Financial difficulty	0 (0–100)	0 (0–66.7)	0.098

**Table 3:** The relationship between TNM stage and clinico-pathological characteristics and quality of life in patients with gastro-oesophageal cancer (*n*=149)

	**TNM I (*n*=28)**	**TNM II (*n*=46)**	**TNM III (*n*=34)**	**TNM IV (*n*=41)**	***P*-value**
Age: (<65 years/ 65–74 years/⩾75 years)	15/12/1	23/21/2	18/9/7	18/13/10	0.482
Sex: (male/female)	17/11	33/13	20/14	31/10	0.387
Tumour type: (squamous/adeno)	2/26	13/33	6/28	4/37	0.576
Tumour site: (oesophagus/gastric)	6/22	33/13	16/18	13/28	0.528
Tumour length: (<5 cm/5–10 cm/>10 cm)	19/7/0	21/22/3	10/19/3	8/22/6	<0.001
Weight loss: (yes/no)	14/14	29/17	23/11	34/7	0.004
Dysphagia score: (1/2/3/4/5)	22/3/3/0/0	20/10/9/7/0	14/5/9/5/1	23/5/10/3/0	0.130
ECOG: (0–1/2/3–4)	22/6/0	33/11/2	27/6/1	24/15/2	0.099
Treatment: (operable/inoperable)	25/3	26/20	15/19	1/40	<0.001
					
EORTC QLQ-C30 (0–100)	Median (range)	Median (range)	Median (range)	Median (range)	
Physical functioning	93.3 (73.3–100)	100 (66.7–100)	100 (73.3–100)	86.7 (66.7–100)	0.023
Role functioning	66.7 (0–100)	66.7 (0–100)	66.7 (0–100)	50 (0–100)	0.058
Emotional functioning	66.7 (8.3–100)	75 (25–100)	83.3 (0–100)	58.3 (0–100)	0.042
Cognitive functioning	83.3 (50–100)	83.3 (33.3–100)	83.3 (16.7–100)	75 (0–100)	0.042
Social functioning	100 (33.3–100)	83.3 (0–100)	66.7 (0–100)	50 (0–100)	0.002
Global quality of life	66.7 (8.3–100)	66.7 (0–100)	50 (16.7–100)	41.7 (0–100)	0.001
Fatigue	27.8 (0–66.7)	22.2 (0–88.9)	33.3 (0–100)	55.6 (0–100)	0.002
Nausea and vomiting	16.7 (0–100)	0 (0–100)	16.7 (0–100)	16.7 (0–100)	0.553
Pain	16.7 (0–66.7)	16.7 (0–100)	33.3 (0–100)	16.7 (0–100)	0.098
Dyspnoea	16.7 (0–100)	0 (0–100)	0 (0–100)	33.3 (0–100)	0.014
Sleep disturbance	(0–100)	33.3 (0–100)	33.3 (0–100)	66.7 (0–100)	0.689
Appetite loss	0 (0–100)	33.3 (0–100)	33.3 (0–100)	66.7 (0–100)	<0.001
Constipation	33.3 (0–66.7)	16.7 (0–100)	0 (0–100)	33.3 (0–100)	0.013
Diarrhoea	0 (0–100)	0 (0–100)	0 (0–33.3)	0 (0–66.7)	0.601
Financial difficulty	0 (0–66.7)	0 (0–100)	0 (0–66.7)	0 (0–100)	0.306

**Table 4:** The relationship between appetite loss, clinico-pathological characteristics and quality of life in patients with gastro-oesophageal cancer (*n*=152)

	**Not at all (*n*=55)**	**A little (*n*=43)**	**Quite a bit (*n*=26)**	**Very much (*n*=28)**	***P*-value**
Age: (<65 years/ 65–74 years/⩾75 years)	27/18/10	18/15/10	8/15/3	13/8/7	0.540
Sex: (male/female)	43/12	26/17	15/11	20/8	0.312
Tumour type: (squamous/adeno)	7/48	5/38	7/19	6/22	0.138
Tumour site: (oesophagus/gastric)	25/30	17/26	15/11	13/15	0.603
Tumour length: (<5 cm/5–10 cm/ >10 cm)	27/20/4	18/21/2	9/15/2	6/14/4	0.016
TNM stage: (I/II/III/IV)	16/17/13/8	8/17/9/9	3/8/7/8	1/4/5/16	<0.001
Weight loss: (yes/no)	26/29	27/16	22/4	26/2	<0.001
Dysphagia score: (1/2/3/4/5)	37/10/6/2/0	24/6/10/3/0	9/4/9/3/1	11/3/7/7/0	<0.001
ECOG: (0–1/2/3–4)	39/14/2	34/8/1	18/7/1	17/9/2	0.281
Treatment: (operable/inoperable)	35/20	20/23	11/15	3/25	<0.001
					
EORTC QLQ-C30 (0–100)	Median (range)	Median (range)	Median (range)	Median (range)	
Physical functioning	100 (73.3–100)	100 (73.3–100)	86.7 (66.7–100)	80 (66.7–100)	<0.001
Role functioning	100 (0–100)	66.7 (0–100)	58.3 (0–100)	33.3 (0–100)	<0.001
Emotional functioning	75 (73.3–100)	66.7 (0–100)	83.3 (8.3–100)	58.3 (0–96.7)	0.003
Cognitive functioning	83.3 (16.7–100)	83.3 (0–100)	83.3 (50–100)	66.7(0–100)	0.001
Social functioning	100 (0–100)	83.3 (0–100)	66.7 (0–100)	50 (0–100)	<0.001
Global quality of life	66.7 (16.7–100)	50 (0–100)	45.8(16.7–100)	29.1 (0–66.7)	<0.001
Fatigue	11.1 (0–88.9)	33.3 (0–83.2)	33.3 (0–100)	77.7 (22.2–100)	<0.001
Nausea and vomiting	0 (0–100)	16.7 (0–100)	16.7 (0–100)	41.7 (0–100)	<0.001
Pain	16.7 (0–100)	16.7 (0–83.3)	33.3 (0–100)	25 (0–100)	<0.001
Dyspnoea	0 (0–66.7)	0 (0–100)	0 (0–100)	50 (0–100)	<0.001
Sleep disturbance	0 (0–100)	33.3 (0–100)	33.3 (0–100)	33.3 (0–100)	0.044
Constipation	0 (0–100)	33.3 (0–100)	33.3 (0–100)	66.7 (0–100)	<0.001
Diarrhoea	0 (0–100)	0 (0–66.7)	0 (0–100)	0 (0–100)	0.512
Financial difficulty	0 (0–66.7)	0 (0–100)	0 (0–100)	0 (0–100)	0.296

**Table 5:** The relationship between systemic inflammatory response, as evidenced by elevated C-reactive protein, clinico-pathological and quality of life characteristics in patients with gastric-oesophageal cancer (*n*=94)

	**CRP<10 (*n*=57)**	**CRP>10 (*n*=37)**	***P*-value**
Age: (<65 years/65–74 years/⩾75 years)	34/16/7	18/12/7	0.258
Sex: (male/female)	38/19	27/10	0.520
Tumour type: (adeno/squamous)	48/9	30/7	0.695
Tumour site: (oesophagus/gastric)	23/34	20/17	0.195
Tumour length: (<5 cm/5–10 cm/>10 cm)	35/17/2	11/20/3	0.005
Tumour stage: (I/II/III/IV)	15/20/13/8	4/10/10/13	0.006
Weight loss: (yes/no)	31/16	27/10	0.072
Dysphagia score: (1/2/3/4/5)	29/14/11/3/0	18/6/10/2/1	0.390
ECOG: (0–1/2/3–4)	52/5/0	27/10/0	0.019
Treatment: (operable/inoperable)	40/17	8/29	<0.001
			
EORTC: (0–100)	Median (range)	Median (range)	
Physical functioning	100 (73–100)	86.7 (66.7–100)	0.001
Role functioning	66.7 (0–100)	66.7 (0–100)	0.040
Emotional functioning	66.7 (0–100)	70.8 (0–100)	0.343
Cognitive functioning	83.3 (16.7–100)	83.3 (33.3–100)	0.875
Social functioning	83.3 (0–100)	66.7 (0–100)	0.045
Global quality of life	66.7 (0–100)	50 (0–100)	0.068
Fatigue	33.3 (0–100)	44.4 (0–88.9)	0.003
Nausea and vomiting	16.7 (0–100)	16.7 (0–100)	0.152
Pain	16.7 (0–100)	16.7 (0–100)	0.040
Appetite loss	33.3 (0–100)	66.7 (0–100)	0.001
